# The Principle and Application of Achromatic Metalens

**DOI:** 10.3390/mi16060660

**Published:** 2025-05-30

**Authors:** Runsheng Liu, Lihua Li, Jian Zhou

**Affiliations:** 1 Guangdong-Hongkong-Macao Critical Optical Components Precision Manufacturing Research Center, Sino German College of Intelligent Manufacturing, Shenzhen Technology University, Shenzhen 518118, China; 2400261010@stumail.sztu.edu.cn; 2 School of Mechanical Engineering, Hefei University of Technology, Hefei 230009, China

**Keywords:** achromatic metalenses, application of metalenses

## Abstract

Metalenses, as ultrathin planar optical devices based on metasurfaces, have attracted significant research interest in recent years due to their compact structure and versatile light manipulation, showing great potential to replace traditional lenses in specific applications. This review focuses on the fundamental principles of geometric and propagation phases of metalenses, introducing their applications in achromatic aberration correction, and emphasizing their advantages and limitations. We further discuss the application of multilayer metalenses in zoom optical systems and summarize methods such as topology optimization and inverse design to enhance the efficiency of metalenses. Special attention is given to comparing broadband and discrete achromatic correction, highlighting their respective working principles, design challenges, and practical implications. Additionally, recent advances in using deep learning for image-side aberration correction are briefly discussed. Finally, we highlight various practical applications of metalenses and discuss future research directions.

## 1. Introduction

Metalenses, a class of two-dimensional optical lenses derived from metasurfaces, have emerged in recent years as promising alternatives to conventional optical systems. Recent research has demonstrated that metalenses exhibit fundamental imaging capabilities. In this paper, we first review the fundamental principles of metalens, with a detailed discussion of both the geometric phase and propagation phase mechanisms. Furthermore, summarizes the core concepts and recent advances in several mainstream designs of achromatic metalenses and illustrates their potential application scenarios through specific examples.

Metalenses are fabricated through advanced metamaterial design by arranging various meta-atoms to create the precise phase profile required for focusing. Compared to conventional lenses, metalenses offer advantages such as ultra-thin profiles, compactness, and reduced weight. Recent studies have also demonstrated that metalenses can be engineered to achieve achromatic performance over specific spectral ranges, highlighting their potential to replace conventional optical components in certain applications.

Depending on the desired functionality and operating wavelength, different materials are utilized in the design and fabrication of meta-atoms. Silicon (Si) [1,2,3], including both crystalline (c-Si) and amorphous (a-Si) forms [4,5,6,7], is commonly employed in the infrared regime, although its application in the visible spectrum [8] has also been investigated. For visible wavelengths, materials such as titanium dioxide (TiO_2_) [9,10,11,12] and silicon nitride (SiN) [13,14,15] are typically selected due to their favorable optical properties.

To reduce the manufacturing cost of metalenses, advanced precision manufacturing techniques such as nanoimprint lithography (NIL) [16,17,18,19,20] and femtosecond laser printing have been increasingly applied in recent years. Although metalenses offer unparalleled advantages in light manipulation and compactness compared to traditional lenses, their widespread application in practical imaging still faces several challenges. These challenges include limitations in fabrication technology, constraints in achromatic bandwidth, and issues related to size. Additionally, high costs, long production times, and stringent precision requirements in electron beam lithography further hinder their large-scale commercialization. The subsequent sections of this paper will provide a detailed discussion of the obstacles hindering the practical application of metalenses. The ultimate goal of optical lenses in the visible light spectrum is to produce high-quality images. Traditional lenses suffer from chromatic aberrations, which often require the use of multiple lens elements or aspherical surfaces to correct. Achromatic metalenses show the potential to improve imaging quality, among other aspects. This paper begins with the fundamental principles of metalenses, summarizes mainstream approaches for achieving achromatic performance, discusses their advantages and disadvantages, and introduces novel applications.

## 2. The Principle of Metalens

In 2011, the research group led by Professor Capasso proposed the generalized Snell’s law [21], significantly enhancing the design flexibility of optical components. Since then, metasurfaces have gradually become a key area of focus in optical research.

(1) $$\left\{ \begin{array}{cc} n_{t}sin\left(\theta_{t}\right)-n_{i}sin\left(\theta_{i}\right) & =\frac{\lambda_{0}}{2\pi }\frac{d\phi \left(x\right)}{dx}\\ n_{i}sin\left(\theta_{r}\right)-n_{i}sin\left(\theta_{i}\right) & =\frac{\lambda_{0}}{2\pi }\frac{d\phi \left(x\right)}{dx} \end{array}\right.$$

The generalized laws of refraction and reflection are given by Equation (1). Here, $n_{i}$ and $n_{t}$ denote the refractive indices of the incident and transmitted media, respectively. $\theta_{i}$, $\theta_{t}$ and $\theta_{r}$ represent the angles of incidence, transmission, and reflection, measured with respect to the surface normal. The term $\frac{d\phi \left(x\right)}{dx}$ corresponds to the spatial phase gradient imposed by the metasurface. This law indicates that when light passes through an object with a phase gradient, its propagation direction is determined not only by the refractive index and angle of incidence but also by the phase gradient. It is important to note that, although differentiability in mathematics requires a function to be continuous over its domain, the phase gradient as defined in Equation (1) is typically discontinuous in practical metalens implementations due to the discretization of the phase profile.

**Figure 1:**
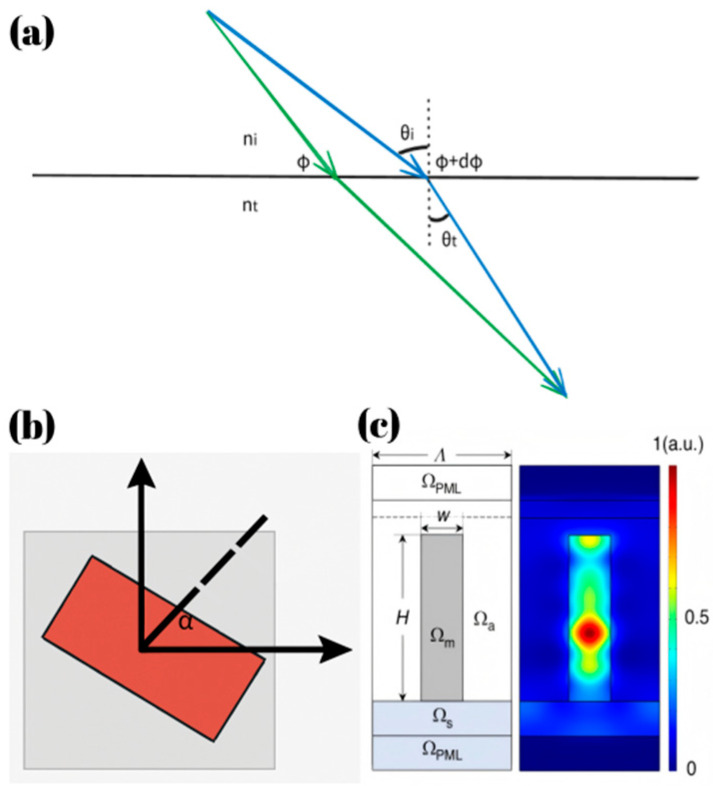
(**a**) Schematic diagram of light propagation through a surface with a phase gradient. (**b**) The meta-atom using PB phase. (**c**) Propagation of light within meta-atoms [29].

In article [21], the Pancharatnam-Berry (PB) phase, also known as the geometric phase, is used in the design of polarization-sensitive metasurface. When polarized light propagates through anisotropic meta-atoms, the rotation of these units induces an additional phase shift. As shown in Figure 1b, by controlling the rotation angle θ, a phase modulation of 2θ is introduced, which is independent of wavelength and depends solely on the rotation angle. This effect causes the transmitted wave to be decomposed into two orthogonal polarization components: the cross-polarized component acquires an extra phase shift of 2θ, while the co-polarized component retains its original phase and is typically considered undesired in PB phase designs.

The propagation phase is another fundamental mechanism in metalens design. As shown in Figure 1c, in the theory of propagation phase, meta-atoms are treated as waveguides. Since light propagates through different optical paths in waveguides with varying parameters, these variations in optical path length can be utilized to control the phase of light, ultimately enabling precise manipulation of light propagation. The phase change when light propagates through a meta-atom can be expressed as: $\begin{array}{c} \varphi =k_{0}n_{eff}h \end{array}$, where $n_{eff}$ is the effective refractive index (By changing the duty cycle of the meta-atom in a basic design unit which is typically achieved by altering the shape or changing the geometric parameters, the effective refractive index can be modified), and h is the height of the meta-atom. For a single wavelength, $k_{0}$ is a constant, while $n_{eff}$ and h are variable parameters. To achieve focusing at a specific wavelength, it is necessary to adjust $n_{eff}$ and h such that the phase variation covers a full 2π range and satisfies the focusing formula, choose meta-atoms exhibiting higher transmittance, thus enabling the desired phase design. In recent research, this method has been predominantly utilized for designing metalenses operating in the visible and infrared bands [22,23,24,25,26,27,28].

## 3. Achromatic Metalens

When a beam of white light passes through a lens, dispersion (the wavelength-dependence of refractive index) causes different colors to refract by different amounts, so they focus at different distances. Traditional optical lens systems primarily achieve achromatic effects by using compound lenses, such as achromatic doublets [30,31,32,33]. An achromatic doublet consists of two different optical materials (typically crown glass and flint glass), leveraging their distinct dispersion properties to compensate for chromatic aberration. Crown glass has a high Abbe number, while flint glass has a low Abbe number. By carefully designing the curvature, thickness, and alignment of the lenses, the chromatic dispersion of each material can be engineered to counteract the other. This design enables the doublet to focus different wavelengths of light more closely together, thereby reducing chromatic aberration within a targeted spectral range. Additionally, traditional optical systems can further improve performance by optimizing the design of lens groups, such as triplet lenses or more complex multi-lens systems. In some high-end applications, diffractive optical elements are integrated into hybrid lens systems to exploit their inherent negative dispersion—counteracting the positive dispersion of refractive components—to effectively reduce chromatic aberration over a broader wavelength range [34,35,36,37,38,39]. Although traditional methods have achieved significant success in chromatic aberration correction, the large size and heavy weight limit their application in certain modern optical systems. The metalenses employ precise designs and offer the advantages of lightweight, and compact size while achieving achromatic performance, providing new directions for the development of future optical systems.

Unlike traditional lenses, metalenses can reduce chromatic aberration by optimizing the material of meta-atoms and substrate, parameters and distribution of meta-atoms. As summarized in Table 1, such metalenses are designed to function across different spectrums of wavelengths. It is evident that there are multiple material options for fabricating achromatic metalenses, and it is crucial to choose appropriate materials and fabrication methods based on different wavelength ranges and functions.

Currently, the primary methods for eliminating chromatic aberration in dielectric metalenses can be categorized into two approaches. The first method is to design metalenses that are inherently capable of achieving achromatic performance. The second method builds upon the first approach by incorporating deep learning to perform chromatic aberration correction during imaging. Regardless of the method, these approaches rely on the phase distribution formula:

(2) $$\varphi \left(r,\omega \right)=-\frac{2\pi }{\lambda }\left(\sqrt{r^{2}+f^{2}}-f\right)$$

**Figure 2:**
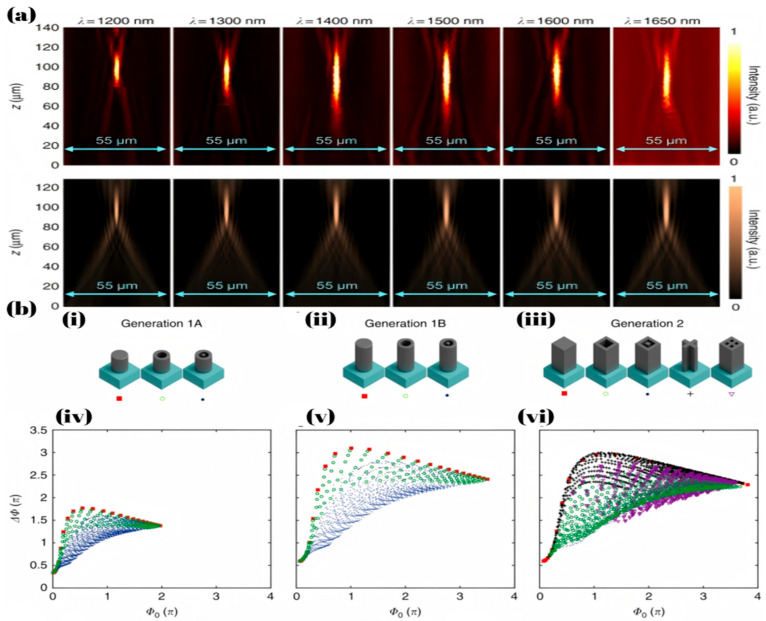
(**a**) Experimental (**first row**) and simulated (**second row**) intensity profiles of the broadband achromatic metalens along the z-plane at various wavelengths [43]. (**b**) Schematic diagrams of various meta-atoms with different geometric parameters (e.g., height, shape, etc.), and the phase and dispersion distribution of corresponding meta-atoms (e.g., singular pillars, annular pillars, crosses, etc.) [44].

The f is the focal length of the metalens, and r denotes the radial position of the metalens. This formula indicates that the phase distribution required for different wavelengths to focus at the same position is different. The cause of chromatic aberration is that the phases generated by different wavelengths after passing through meta-atoms cannot match the phase required for focusing, which leads to differences in the focal positions. Although the phase response exhibited by a metalens is inherently wavelength-dependent, precise phase compensation techniques can be employed to effectively correct the phase discrepancy between the target focalization requirements at different wavelengths and the phase profiles provided by meta-atoms. Wang, S et al. [43] proposed decomposing the phase into the phase corresponding to the longest wavelength and a compensatory phase, as shown in Equation (3), where the former is provided by the geometric phase and the second by the resonant phase, achieving the focusing performance as shown in Figure 2a. These two methods of phase generation are based on different principles, so these two phases do not affect each other.

(3) $$\varphi_{Lens}\left(r,\lambda \right)=\varphi \left(r,\lambda_{max}\right)+\Delta \varphi \left(r,\lambda \right)$$

As shown in Equation (3), $\varphi \left(r,\lambda_{max}\right)$ is a fixed phase component that depends only on the specified maximum wavelength and remains invariant across different wavelengths (the phase generated by geometric phase, the rotation angle θ = $-(\frac{\pi }{\lambda_{\max}}\sqrt{r^{2}+f^{2}}-f)$). $\Delta \varphi \left(r,\lambda \right)$ is the compensation component, different wavelengths correspond to a compensation term, through the combination of these two items so that the phases required for different wavelengths can match, thus achieving achromatic aberration. The phase difference between different wavelengths and the maximum wavelength can be expressed by Equation (4).

(4) $$\Delta \varphi \left(r,\lambda \right)=-\left[2\pi \left(\sqrt{r^{2}+f^{2}}-f\right)\right]\left(\frac{1}{\lambda }-\frac{1}{\lambda_{max}}\right)$$

When the phase compensation satisfies the above equation, achromaticity is achieved within a specific range. The article [43] uses the geometric phase to achieve a fixed phase change, those types of metalenses are polarization-sensitive [45,46,47]. Achieving polarization-independent metalenses requires the use of the propagation phase method, which in turn necessitates highly symmetrical meta-atom designs. As shown in Equation (5), the phase required for focusing can be expanded as a Taylor series. The first term represents the phase at the central frequency, and the second term corresponds to the group delay ($\frac{d\varphi \left(r\right)}{d\omega }{|}_{\omega =\omega_{0}}$), and the third term represents the group delay dispersion ($\frac{d^{2}\varphi \left(r\right)}{2d\omega^{2}}{|}_{\omega =\omega_{0}}$).

(5) $$\varphi \left(r,\omega \right)=\varphi \left(r,\omega_{0}\right)+\frac{d\varphi \left(r\right)}{d\omega }{|}_{\omega =\omega_{0}}\left(\omega -\omega_{0}\right)+\frac{d^{2}\varphi \left(r\right)}{2d\omega^{2}}{|}_{\omega =\omega_{0}}{\left(\omega -\omega_{0}\right)}^{2}+\cdots$$

In the majority of studies, the first two terms in Equation (5) are predominantly incorporated. Shrestha, S et al. [44] Proposed a dielectric metalens which achieved achromatic performance by setting the first two terms to meet the focusing requirements. This metalens achieving 50% focusing efficiency with continuous broadband achromatic performance over the 1200 nm to 1650 nm wavelength range has been demonstrated.

To achieve better achromatic performance, in recent years researchers have designed corresponding meta-atoms shape or improved the position distribution of the meta-atoms on the metalens by establishing different phase distribution formulas. Sun, X. [48] et al. established a phase compensation formula: $\Delta \varphi^{\prime }\left(x_{i},y_{i},\lambda \right)=\Delta \varphi \left(x_{i},y_{i},\lambda \right)+\frac{\alpha }{\lambda }+\beta$, which achieves a metalens with an average focusing efficiency of 36.4%, numerical aperture (NA) of 0.564, which operating band ranging from 530 nm to 850 nm. Balli, F et al. [49] utilized specially designed meta-atom shapes combined with a phase plate, setting phase distributions as $\phi \left(r\right)=\frac{\pi \phi_{0}^{’}}{2}\left(2r\sqrt{1-\frac{4r^{2}}{EPD^{2}}}+EPD\cdot \arcsin(\frac{2r}{EPD})\;\right),\;\mathrm{entrance}\;\mathrm{pupil}\;\mathrm{diameter}\;\left(\mathrm{EPD}\right)$, achieving an achromatic metalens with an NA of 0.27, an average focusing efficiency of 60%, and a focal length error of less than 6%. Li, J. et al. [50] optimized the light propagation geometry from a point source through the metalens to design a phase profile that corrects both chromatic and spherical aberrations simultaneously, achieving a metalens with an NA of 0.635 and achromatic performance in the 490 nm to 720 nm range.

Nevertheless, no matter how the phase distribution is optimized, the size and other parameters of the achromatic metalens are still subject to certain limitations in broadband achromatic metalens. As shown in Equation (6) [44], the NA, radius and achromatic bandwidth of the metalens are mutually constrained, meaning that changing one of these values will inevitably limit the other two. This trade-off arises because, as the metalens size increases and a broader achromatic bandwidth is required, the necessary phase compensation range also expands; As shown in Figure 2b, the use of a more extensive meta-atom library enabled an expanded phase dispersion space. However, these compensation methods have inherent limitations.

(6) $$R_{max}\leq \frac{\Delta \Phi^{\prime }c}{\Delta \omega \left(\frac{1}{NA}-\sqrt{\frac{1}{NA^{2}}-1}\right)}$$

To expand the range of phase compensation, researchers typically select suitable materials or design meta-atoms with higher aspect ratios. However, both approaches face significant challenges. Firstly, high-refractive-index materials, such as silicon, exhibit low efficiencies in the visible and other spectral ranges, and materials that have both high efficiency and high refractive index across a broad bandwidth are relatively rare. To mitigate this effect, Yoon, G. et al. [51] added TiO_2_ nanoparticles to a UV-curable resin matrix. By using this high-refractive-index nanocomposite, they achieved the fabrication of low-cost metalens with a focusing efficiency of 33% at 532 nm through ultraviolet NIL. Secondly, taller meta-atoms correspond to higher aspect ratios, which place greater demands on fabrication techniques. Excessively high aspect ratios may lead to the Aspect Ratio Dependent Etching (ARDE) effect, resulting in sidewall tapering and introducing phase errors that degrade the metalens performance [52]. Wang, Y. et al. [53] successfully fabricated two broadband achromatic metalenses with NA of 0.1 and 0.24, a radius of 25 µm and 30 µm, with a focal length variation of less than 7% and an average focusing efficiency of 77.1–88.5% in the wavelength range of 650 nm to 1000 nm. Due to the relatively large phase compensation required in the broadband, they used ${\mathrm{TiO}}_{2}$ as the material to fabricate meta-atoms with a height of 1500 nm, which is one of the highest aspect ratios in available meta-atoms.

As shown in Equation (6), the parameters of such metalens are constrained to each other, so how to properly design the parameters of metalens in different application scenarios is an important issue. As a computational design methodology, topology optimization systematically arranges materials to design meta-atoms with optimal performance under given constraints.

In topology optimization, the design domain of a meta-atom is divided into multiple sub-regions or grids, and material distribution in each grid is optimized to maximize a given objective function, such as transmission efficiency or phase accuracy. Zhang, L et al. [54] optimized meta-atoms to achieve the desired group delay and maximum polarization transformation efficiency by using topology optimization, achieving an average focusing efficiency of 53% in the visible light spectrum. Zheng, Y. et al. [55] utilized topology optimization to design a metalens with an average depth of focus (DOF) of 18.80 μm and an average focusing efficiency of 72.57%. Wei, Y. et al. [56] applied topology optimization to enhance the cross-polarization conversion efficiency of meta-atoms, achieving an average cross-polarization conversion efficiency of 88.5%.

In addition to topology optimization, other function optimization methods can also be applied to the performance optimization of metalens. By maximizing the minimum light intensity (MMI) at the focal point for different wavelengths, Jiang, Q et al. [57] achieved discrete achromaticity in the visible light spectrum using an inverse design framework, with a focal length variation range of 5.28%.

Additionally, deep learning can be employed for inverse design or optimize the shapes of meta-atoms to meet the required phase or transmittance specifications. Wang, F et al. [58] trained a deep neural network (DNN) to quickly predict the optical responses (amplitude, phase, transmittance, etc.) of meta-atoms, significantly reducing the computational time required for simulations. Zhang, C et al. [59] significantly improved the design efficiency and enhanced the achromatic capability of the metalens by combining PNN with Particle Swarm Optimization and Genetic Algorithm. Hwang, S et al. [60] designed a large-dimensional neutron-focusing metalens using a diffractive deep neural network (D2NN) based on the Rayleigh-Sommerfeld diffraction theory. Wu, H et al. [61] proposed an inverse design system that includes a forward simulator and a global optimization model, enabling rapid prediction of the optical response of meta-atoms using DNNs. Xu, S. et al. [62] proposed an efficient inverse design framework for metalenses, incorporating a forward prediction network and a global multi-objective optimization model. Using this framework, they designed an achromatic metalens operating in the mid-infrared range, achieving an average focusing efficiency of 61.37% and a maximum focal length deviation of 3.56%.

The previously mentioned metalenses achieve achromatic performance by adjusting the parameters or distribution of the meta-atoms, which in turn modulate the phase profile for different wavelengths passing through the metalens. From the design process, it can be inferred that once such a metalens is designed, its optical properties are also defined. For conventionally designed single-layer metalens, parameters such as the focal length and NA are fixed. However, the emergence of multi-layer metalens has expanded the application scenarios. This type of metalens system can change functions such as focal length by altering the relative angles or lateral distances between each layer. In addition, this type of metalens is capable of providing enhanced phase compensation, thus enabling achromatic performance over a broader wavelength range and holds the potential to overcome the limitations among various parameters described in Equation (6). Yu, H. et al. [63] defined the objective function $max(\sum_{\lambda }\sum_{\theta }\eta_{\lambda ,\theta }\left(r_{atom}\left({\vec{x}}_{1}\right),\alpha_{atom}\left({\vec{x}}_{2}\right)\right)$, in order to maximize the focusing efficiency of the metalens. By cascading two layers of metalenses, they used inverse design to optimize the radius of the meta-atoms in the first layer and the corresponding rotation angles of the meta-atoms in the second layer, achieving an achromatic metalens with a wavelength range of 720 nm to 780 nm. Che, X. et al. [64] integrated Double-layer metalens into a variable focal length system. By changing the relative position between the two layers of metalens, the function of variable focal length is realized. The variable focal length metalens designed by this method will change the corresponding effective NA when the focal length is changed, and the corresponding phase distribution of this system is shown in Equation (7).

(7) $$\begin{array}{cc} \varphi_{1}\left(x,y,\lambda \right) & =\frac{\pi x^{3}\left(f_{2}-f_{1}\right)}{6d_{max}\lambda f_{1}f_{2}}+\frac{\pi xy^{2}\left(f_{2}-f_{1}\right)}{2d_{max}\lambda f_{1}f_{2}}-\frac{\pi \left(x^{2}+y^{2}\right)}{2f_{1}\lambda }\\ \varphi_{2}\left(x,y,\lambda \right) & =\frac{\pi x^{3}\left(f_{1}-f_{2}\right)}{6d_{max}\lambda f_{1}f_{2}}+\frac{\pi xy^{2}\left(f_{1}-f_{2}\right)}{2d_{max}\lambda f_{1}f_{2}}-\frac{\pi \left(x^{2}+y^{2}\right)}{2f_{1}\lambda } \end{array}$$

Through the precise design of phase distribution, it can achieve an achromatic effect and simultaneously adjust the focal length. It should be noted that the initial position of meta-atoms in such metalenses, particularly the relative alignment between the two layers, must be set with high precision. Even a small displacement may alter the phase, thereby affecting both the achromatic aberration and the focal length adjustment.

Multilayered metalens can also be equated to metalens with higher meta-atoms, representing their ability to provide larger phase compensation, which exists as a potential application for metalens with large NA. Yao, Z et al. [65] used Si and TiO_2_ to fabricate meta-atoms, via layer-by-layer deposition and lithography techniques, to construct double-layer metalens. This approach reduces the aspect ratio to 3 by stacking high-refractive-index materials (for comparison, the aspect ratio of traditional single-layer materials in the visible spectrum is typically greater than 6). Feng, W et al. [66] fabricated double-layer RGB achromatic metalens by electron beam lithography using c-Si and Si_3_N_4_ as meta-atom materials. This design provides a higher degree of vertical freedom and reduces the difficulty of designing meta-atoms, which achieve RGB achromatic metalens by using two types of meta-atoms: cylindrical and rectangular columns.

**Figure 3:**
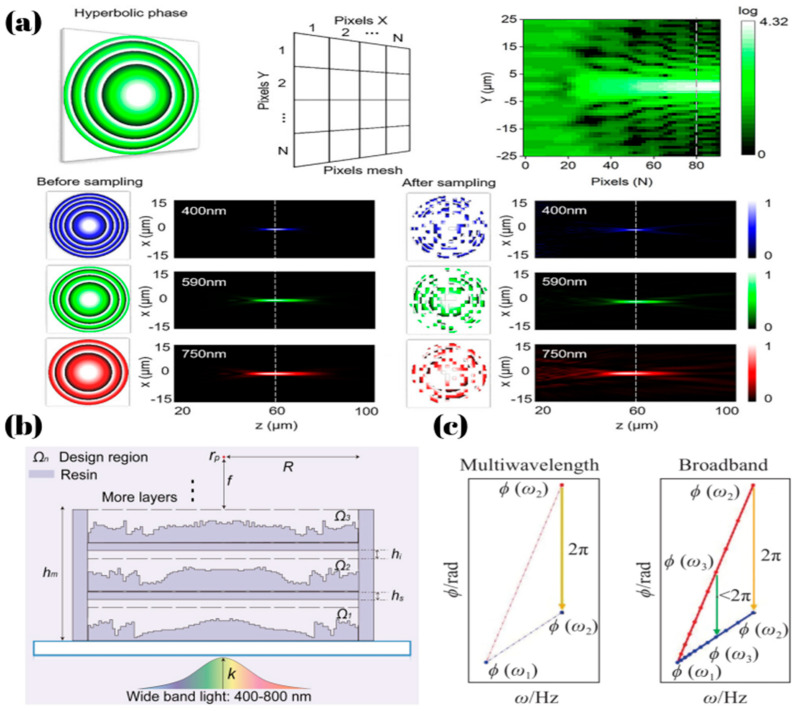
(**a**) The hyperbolic phase distribution. The schematic diagram of sampling mesh on the diffractive surface. The optical field distribution with hyperbolic phases at operating wavelengths of 400 nm, 590 nm, and 750 nm. The optical field distribution after random sampling at corresponding wavelengths [68]. (**b**) Schematic of the design model and optimization region with indicated parameters described in the text, and deconstructed MAM showing single-layer, double-layer, and triple-layer (full) structures [67]. (**c**) The differences between discrete multiwavelength metalenses and broadband metalenses in terms of phase processing [69].

Although multilayer metalenses offer a broader dispersion compensation range, they are more difficult and expensive to fabricate compared to single-layer metalenses. This is because these metalenses require precise alignment of multiple layers to ensure that the total phase distribution aligns with the desired design. However, precisely aligning different layers of metalenses is challenging due to the small size of meta-atoms (typically in the nanometer range), and thus misalignment is inevitable when working at such a small scale. Such small positional errors can alter the total phase distribution, thereby affecting the performance of the metalens. As shown in Figure 3b, Pan, C.-F. et al. [67] employed topology optimization as an inverse design method to develop a multilayer achromatic metalens using low-refractive index materials. The structure was fabricated by femtosecond laser-based two-photon polymerization 3D printing, resulting in metalens with an NA of 0.5 and 0.7 with broadband achromatic performance in the visible spectrum, where the 0.5NA metalens exhibited a focusing efficiency of 42%.

In addition to spatial multiplexing in the vertical direction, the multiplexing of meta-atom positions can also enhance its performance. Wang, X. et al. [68] proposed a holographic broadband achromatic metalens by designing the phases of three wavelengths. As shown in Figure 3a, the positions of meta-atoms in three channels follow a probabilistic model $(p_{B}:p_{G}:p_{R}$ = 1:1.5:1), achieving discrete achromatic performance in the 470 nm to 690 nm range with a focal length variation of 5.26%.

In achromatic metalens, the previously mentioned multilayer structures or metalenses composed of high aspect ratio meta-atoms are primarily designed to broaden the dispersion compensation range, thereby enabling metalenses with brand achromatic. The reason for the constraint described by Equation (6) is the inherent upper limit of dispersion compensation. For a broadband achromatic metalens operating in the 8–12 μm wavelength range, with a radius of 1000 μm and an NA of 0.45, calculations based on Equation (2) indicate that a phase dispersion compensation of approximately 20π is required. In practice, however, meta-atoms fabricated from Si in this range typically provide a maximum phase compensation range of only 2π to 3π, which is significantly lower than needed for broadband achromatic performance. Therefore, the required phase dispersion compensation range needs to be reduced—this is usually achievable only by decreasing the metalens radius or lowering the NA, which is consistent with the constraint expressed in Equation (6). What’s more, a broader bandwidth requires phase matching across a larger number of wavelengths, which in turn demands a more diverse phase-dispersion library of meta-atoms; otherwise, efficient broadband achromatic performance cannot be achieved. As shown in Figure 3c [69], in broadband metalenses, although the phase profile is designed within the [0, 2π] range, meta-atoms cannot simultaneously satisfy the required phase shifts for multiple wavelengths due to material dispersion and structural limitations, ultimately leading to phase mismatch. Currently, large-scale achromatic metalenses typically employ various optimization algorithms to minimize phase mismatch and other adverse effects, thereby achieving achromatic performance. Algorithms such as MMI, summarized earlier, were proposed based on this principle. Moreover, the trade-off between structural complexity, manufacturing difficulty, and achromaticity always exists in this method. Besides structural size, cost and other factors still have a significant impact on the commercialization of this type of metalens, and there is no better solution available at present. However, the application of deep learning to correct chromatic aberration in metalens imaging has the potential to mitigate these challenges. Deep Learning has been widely applied in traditional imaging [70]. For example, most modern smartphones utilize convolutional neural network-based algorithms to process captured images.

**Figure 4:**
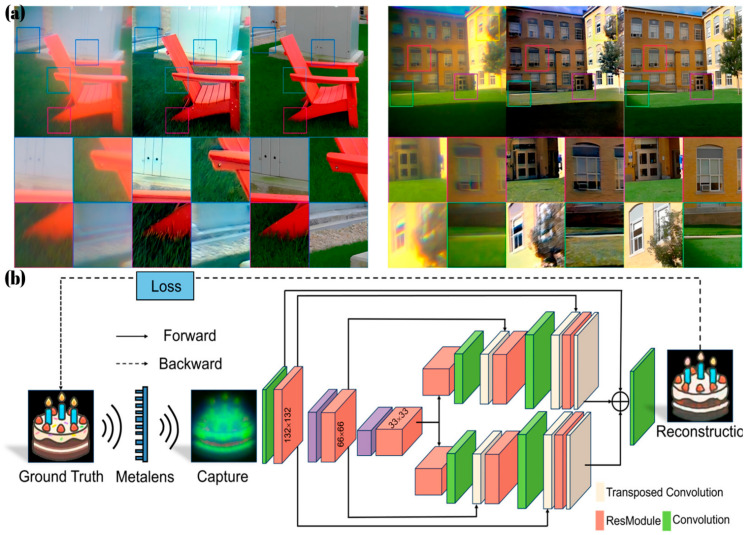
(**a**) The schematic diagram of the chromatic aberration correction process using deep learning [72]. (**b**) The flowchart of the chromatic aberration correction process using deep learning [73].

Additionally, image-generation techniques, which have gained significant attention in recent years, are also based on deep learning. Deep learning can enhance image quality through various methods, such as denoising. Radlak, K et al. [71] developed an Impulse Detection Convolutional Neural Network (IDCNN). Instead of residual learning, the proposed network performs pixel-wise classification to detect impulsive noise, enabling selective restoration of corrupted pixels via a switching filter strategy that preserves uncorrupted image regions. These examples demonstrate that deep learning can significantly improve imaging quality. Therefore, it is feasible to achieve achromatic performance by combining single-wavelength metalenses with deep learning-based training. Dong, Y. et al. [72] used deep learning to correct chromatic aberration and restore RGB channel alignment in imaging of metalens. As shown in Figure 4a, this method effectively enhances color accuracy and detail clarity in real-world images. Cheng, W et al. [73] proposed a minimalist achromatic metalens design based on Optoelectronic Computation Fusion (OCF), effectively solved the chromatic aberration problem of metalenses in imaging applications. They employ only the rectangular shape of meta-atoms, making it highly efficient for simulation and fabrication. By employing deep neural networks to learn the mapping between chromatic-aberrated images and real images, OCF system achieves broadband chromatic aberration correction as shown in Figure 4b. 

Those two types of systems mentioned above, which integrate deep learning, may represent a potential direction for development, as they require only a single wavelength of light as the primary wavelength. The steps to achieve the design of an achromatic metalens system through deep learning are as follows: first, design a metalens capable of focusing at a single wavelength; then, construct an achromatic model using deep learning. After extensive training, an achromatic system for the metalens is successfully realized.

Although the use of deep learning significantly reduces the design complexity, these systems still require training with a large database, meaning that a substantial number of images need to be taken, which requires considerable time. Zhang, Y et al. [74] proposed an imaging model for metalenses based on the Point Spread Function (PSF) through experimental testing. The formula corresponding to the imaging model is as follows: I=O⊗PSF+N. The first term represents the convolution of the object image O with the PSF, and N corresponds to the noise in the system. By using this model and performing certain data calculations, a large training database can be generated, allowing the retrieval of images of various qualities. With this system, high-quality imaging can be achieved using metalens-integrated system. Wei, J. et al. [75] integrated a high-frequency enhancement (HFE) loss module with a bidirectional cyclic generative adversarial network (Cycle-GAN) to develop the HFE Cycle-GAN, which enhance image quality in long-wave infrared metalens imaging. The various achromatic methods presented in this chapter show that metalenses have made significant progress in chromatic aberration correction. However, challenges such as limitations in application scale and operational bandwidth persist. For those large-scale achromatic metalenses, achieving ideal achromatism is difficult due to phase compensation limitations. Those approaches based on deep learning for achromatic correction show potential to address these difficulties, but further research is still required. Although achromatic metalenses currently face certain limitations, they still offer significant advantages in specific applications.

## 4. Application of Metalens

Achromatic metalenses are currently a key focus in metasurface research. For optical systems operating across multiple wavelengths (especially those requiring high resolution), chromatic aberration correction is essential. Owing to their ultrathin profile and powerful wavefront manipulation capabilities, metalenses hold promise for enabling compact, single-lens imaging systems. This is the primary motivation for the detailed discussion of achromatic design strategies in this section. Applications such as super-resolution imaging, miniaturized imaging systems, and augmented reality all demand a certain degree of chromatic correction, underscoring the importance of continued research. Although broadband achromatic performance remains technically challenging, metalenses have already demonstrated excellent functionality in specific applications, and currently attract widespread attention for these use cases.

Chung, H. et al. [76] utilized inverse design to realize a metalens with a transmission efficiency of 85.5%, which enables the development of a high-resolution, high-efficiency maskless lithography system (ZPAL). Kanwal, S et al. [77] realized a metalens operating in the ultraviolet spectrum with polarization insensitivity and FWHM at the focus approaching the diffraction limit, providing a novel approach for miniaturizing devices operating in the ultraviolet spectral range. Zhang, Q. et al. [78] designed a high-NA (NA = 0.97) metalens, operating at a wavelength of 632.8 nm. Under oblique incidence, it achieved super-resolution imaging with a focal spot size smaller than 0.45λ, offering a novel solution for far-field, high-speed super-resolution imaging. Gao, X. et al. [79] designed an all-dielectric bifocal metalens (ADBM) with distinct focal lengths for TM and TE polarization modes (TM: 37.98 mm, TE: 19.93 mm), achieving near-diffraction-limit focusing performance. Building on this, the research team developed a THz imaging system, enabling high-resolution imaging and offering a novel solution for high-resolution imaging and optical communication in the THz domain. Li, Y. et al. [80] employed a water-immersion metalens with a 405 nm laser to generate a super-oscillatory nondiffracting beam with a propagation distance of 60 μm. The beam achieved a transverse resolution of 0.31λ, exceeding the limits of traditional lens systems and offering a novel solution for super-resolution imaging and optical manipulation.

The above examples demonstrate that metalenses are well-suited for applications requiring high resolution. Dai, X. et al. [81] proposed a method to design achromatic super-resolution metalens by wavefront modulation and holographic synthesis. By regarding the achromatic super-resolution metalens as a multiwavelength holographic device, its complex amplitude transmittance function can be expressed as:

(8) $$T\left(x,y,f;\lambda_{1},\ldots ,\lambda_{N}\right)=\sum_{i=1}^{N}c_{i}T_{i}\left(x,y,f;\lambda_{i}\right)$$

**Figure 5:**
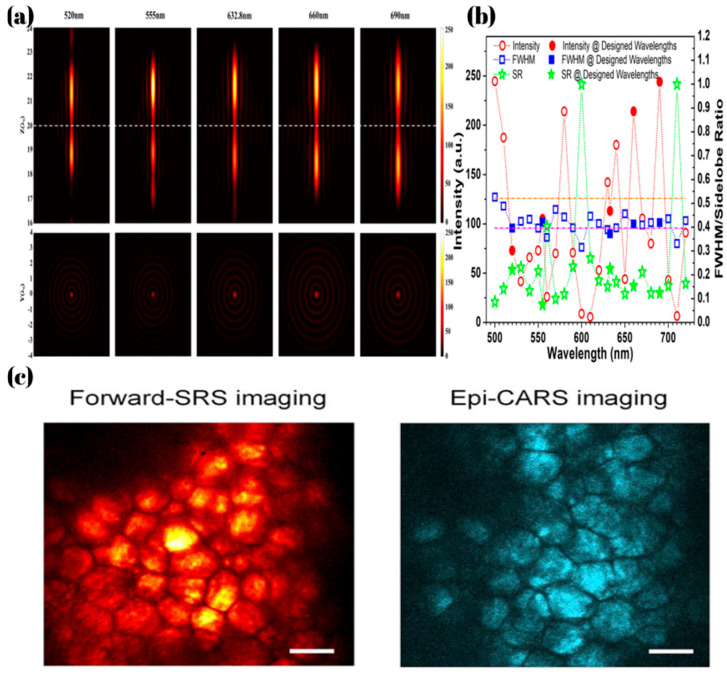
(**a**) The focusing performance of the super-resolution metalens system at different wavelengths [81]. (**b**) Theoretical prediction of the focusing parameters of the achromatic super-resolution lens at different wavelengths, including the peak intensity (red), FWHM (blue), and SR (green), where the results for the five design wavelengths are indicated by solid markers. The Abbe diffraction limit (0.5λ/NA) and the superoscillation criterion (0.38λ/NA) are represented by the orange and violet dash-dot [81]. (**c**) Metalens-based CRS imaging of lipid content in an ex vivo mouse ear. The left image shows the forward SRS image, and the right image shows the epi-detected CARS image [82].

By controlling the weights $c_{i}$, the mutual influence between the screen functions corresponding to different wavelengths can be reduced. This makes the phase generated by different wavelengths after passing through the metalens more closely match the phase required for focusing, thereby endowing the metalens with the functionality of super-resolution achromatism as shown in Figure 5a,b. This design achieves an NA of 0.96 and a diffraction limit of 0.52*λ* at five different wavelengths.

It is expected to be applied in many fields such as biomedicine, microscopic imaging, etc. This type of metalens is applied in the imaging of biological tissues, which is expected to play a role in cancer diagnosis.

In biological imaging, to match the vibrational frequencies required by coherent Raman scattering microscopy, the excitation wavelengths must be precisely tuned to selectively probe specific molecular vibrational modes. Since the operating environment is liquids, Lin, P et al. [82] designed a water-immersion achromatic metalens which works at a wavelength of 800 nm to 1040 nm, achieving a metalens with NA of 0.4 and a wavefront aberration factor (WAF) less than 0.072λ. The CRS microscope system was designed by combining refractive lenses with a metalens. Compared to traditional single-refractive-lens systems, its lateral and axial resolutions were improved by 1.3 times and 3.8 times, respectively.

**Figure 6:**
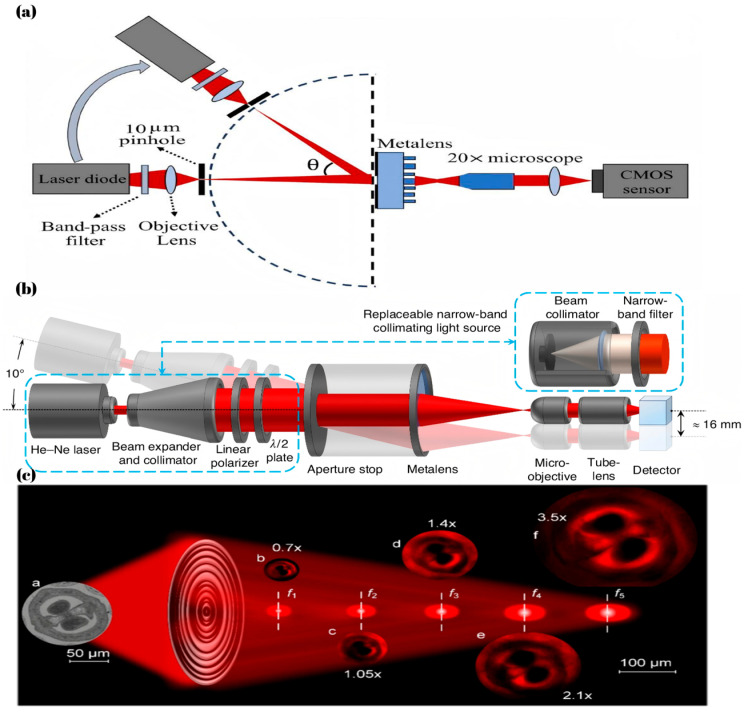
(**a**) The optical path diagram of the wide-field metalens and its imaging performance results [84]. (**b**) The optical path diagram of the wide-field telescope system based on metalens [29]. (**c**) Schematic diagram of a multi-focus metalens with variable magnification [88].

Due to their thin and lightweight nature, metalenses are ideal for integration into miniaturized devices such as endoscopes and handheld microscopes, offering significant promise for improving medical imaging quality. Barulin et al. [83] developed an axially multifocal metalens with an extended DOF of 580 μm and integrated it into a MeD-PAM system, enabling high-resolution, label-free 3D photoacoustic imaging. This work highlights the potential of metalens in biomedical imaging system. Moghaddasi et al. [84] designed an optical system based on near-infrared metalens as shown in Figure 6a, which is optimized for capsule endoscopy. The phase profile was defined as: $\varphi \left(x,y\right)=M\sum_{k=1}^{n}a_{k}{\left(\frac{\rho }{R}\right)}^{2k}$, with coefficients optimized to minimize focal spot size across incident angles, effectively suppressing off-axis aberrations and achieving a wide 165° field of view. The assembled metalens measures 1.58 mm in diameter—much smaller than conventional endoscopes. It demonstrated superior optical performance, with an MTF of 0.3 at 250 lp/mm, offering a compact and high-resolution imaging solution for capsule endoscopy.

Wang, J et al. [29] designed a metalens with a diameter of 46.8 mm and developed a high-resolution portable telescope system with a 20° FOV. They set the phase order to 6 and optimized the width and height of atoms to maximize the focusing efficiency of metalens. Given that the annular meta-atoms respond differently to various polarizations, making the system polarization-sensitive, the study proposed optimizing the phase difference between TE and TM waves, as well as adjusting the meta-atom parameters, to minimize the impact of polarization changes on focusing. Shalaginov, M et al. [85] implemented an infrared imaging system based on a metalens with an ultra-wide field of view (exceeding 170°). Li, S et al. [86] designed a metalens with a working wavelength of 633 nm and an FOV of 100° (The performance of system was experimentally validated at an FOV of 90°). By integrating a deep learning algorithm, the system enables human posture detection and QR code scanning, demonstrating potential applications in vision inspection systems. Engelberg, J et al. [87] utilized a Huygens nanoantenna structure to design a wide-field metalens (FOV ± 15°) operating in the near-infrared spectrum, achieving high-resolution imaging under outdoor conditions. Cao, G et al. [88] developed a 200 nm-thick graphene oxide metalens with five discrete focal lengths (100–500 μm), using spatial multiplexing to minimize inter-focal interference. As shown in Figure 6c, different focal lengths correspond to different magnification ratios, which provides a new idea for the realization of integrated metalens.

In addition to wide-field imaging, single-molecule fluorescence detection plays a significant role in biomedicine due to its high sensitivity and resolution. Barulin, A et al. [89] developed a portable single-molecule detection system using dual-wavelength metalens that focuses 635 nm excitation light and collects 670 nm fluorescence. The metalens achieve high transmittance (82.6% and 83.5%) and conversion efficiency (71.6% and 76.2%). It enables the detection of 1.7 molecules and real-time monitoring of fluorescence bursts from quantum dots and stained nanoparticles, distinguishing particle sizes via diffusion times.

Quantitative phase detection, which indirectly obtains phase information by correlating changes in light intensity with phase variations, is widely used to detect translucent biological cell samples. However, conventional quantitative phase detection systems are complex, and the precise alignment requirements make miniaturization of the equipment challenging. Shanker, A et al. [90] leveraged the intrinsic chromatic dispersion of metalenses, encoding depth information into the spectral domain to design a quantitative phase detection system. By utilizing the relationship between phase variation and wavelength-dependent focal length, they extracted phase information from intensity measurements. A hyperboloid metalens with a 0.5 mm aperture, 1 mm focal length, and NA of 0.25 was fabricated, achieving a focal length shift of 180 μm under 625 nm and 455 nm illumination, closely matching the theoretical value of 190 μm. The system employs spectral multiplexing to separate intensity distributions at different wavelengths, enabling phase retrieval via the transport of intensity equation. This approach allows for high-resolution imaging of phase objects while mitigating the limitations of interferometric methods, demonstrating significant potential for real-time quantitative phase imaging.

Besides these applications, metalenses have also demonstrated great potential in other fields. Chen, J et al. [91] utilized 3D printing technology to fabricate broadband achromatic metalens operating in the 0.2 to 0.9 THz terahertz band. Based on this, they designed a dual-metalens focusing imaging system, achieving super-resolution imaging capabilities. Park, J.-S et al. [92] employed deep-ultraviolet (DUV) lithography to fabricate large-scale metalens with a diameter of 100 mm, consisting of 18.7 billion meta-atoms. Based on this, they designed a compact single-metal imaging system, successfully capturing distant astronomical objects such as sunspots and the Moon. Zhang, L et al. [93] utilized DUV technology to fabricate a metalens with a diameter of 80 mm, operating in the near-infrared band (1450 nm). Based on this, they constructed a single-lens telescope and successfully captured images of the Moon’s surface, demonstrating the potential applications of large-scale metalenses in astronomical observations.

**Figure 7:**
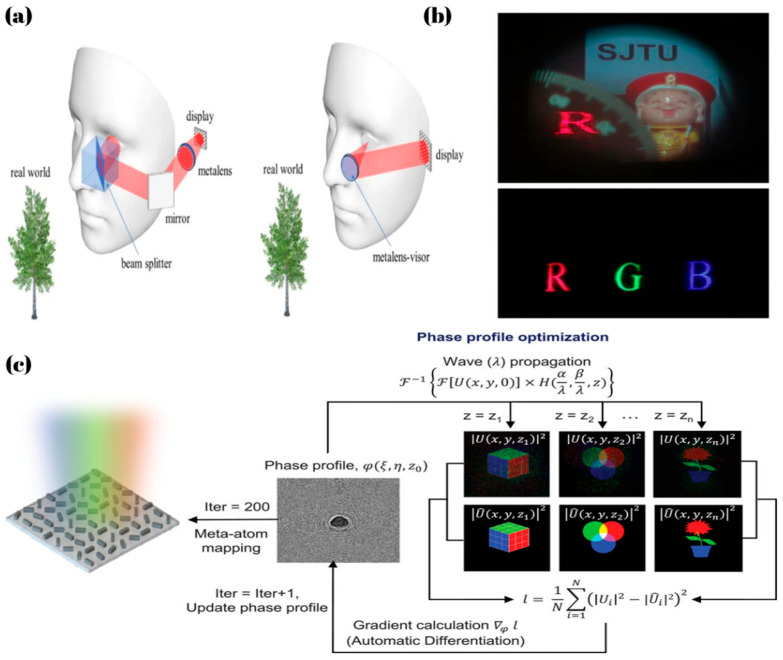
(**a**) The comparison diagram of near-eye AR systems based on traditional transmissive metalens and see-through reflective metalens [94]. (**b**) Imaging results of the near-eye AR system based on see-through reflective metalens [94]. (**c**) Flowchart of the inverse design method for multifunctional metasurface [95].

Augmented Reality (AR) is a technology that overlays virtual information into the real world. Using cameras and sensors, it recognizes the environment in real time and integrates 3D images, text, or sound with the physical surroundings. It is widely used in fields such as education, gaming, and industry. Li, Y et al. [94] designed a dielectric metalens based on PB phase, to replace the off-axis eyepiece and optical combiner in an AR system. As shown in Figure 7a, the system employs a spatial light modulator (SLM) to generate virtual images. By incorporating Maxwellian view display technology, the virtual image of this system remains consistently sharp. This study highlights the potential of metalenses in AR applications by reducing system complexity and improving imaging performance.

Holography achieves 3D imaging by recording and reconstructing a light field’s complete information, utilizing interference and diffraction to present depth and stereoscopic effects. Unlike traditional imaging, it captures both intensity and phase, restoring an object’s true 3D structure. It is widely applied in 3D display, data storage, and optical encryption, with dynamic holography enabling real-time interactive displays. Metasurfaces, capable of controlling light’s phase and amplitude, offer great potential for holography. By arranging meta-atoms to match phase distributions, holographic displays can be realized. Yin, Y. et al. [96] proposed an end-to-end inverse design framework that optimizes the parameters of meta-atoms by minimizing a loss function, successfully realizing a holographic metasurface capable of operating across multiple wavelengths, polarization states, and focal planes. Similarly, S. So et al. [95] realized multiplane holographic display by applying an inverse design approach to optimize the phase distribution. As shown in Figure 7c, they calculated the phase profiles needed to reconstruct distinct images at different lateral positions for each RGB wavelength—with each wavelength forming its image at the same depth but at a different transverse location, and the combination of these images constituting a complete scene. By employing NIL technology, the functionality of displaying holograms at multiple focal lengths was realized, achieving the average efficiency of 63% at RGB wavelengths. Although this method successfully enabled multiplane holography, the static nature of the metasurface remains a key limitation.

**Figure 8:**
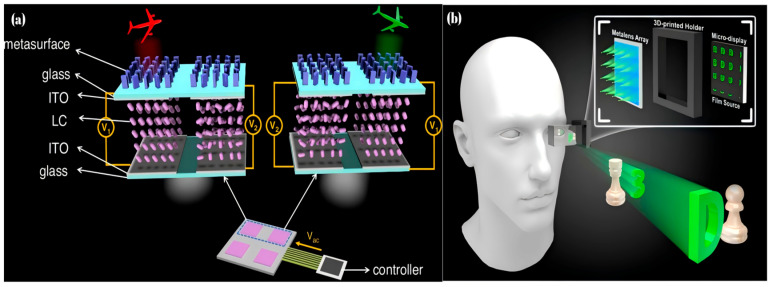
(**a**) Schematic illustration of the electrically tunable PDLC-metasurface dynamic 3D holographic display. CCD-captured 3D holographic images and their corresponding simulated values [97]. (**b**) The 3D AR effect of the meta-II NED with the major components of the metalens array and the micro-display [100].

Sun, S et al. [97] realized a dynamically tunable holographic display by cascading polymer-dispersed liquid crystal (PDLC) with a broadband metasurface. As shown in Figure 8a, in this system, the PDLC layer is precisely controlled in thickness by spherical spacer particles (7 μm to 18 μm in thickness). Combined with electric field control of liquid crystal alignment, the system achieves switching between off-state transmittance below 5% and maximum transmittance exceeding 80%. The dynamic change in transmittance ultimately facilitates the dynamic display of holographic images. Additionally, this system offers higher contrast, and faster response times, and supports multi-channel multiplexing, enabling the display of more complex holographic images. Bosch, M et al. [98] designed a voltage-tunable focusing imaging system by integrating liquid crystals with metalens. Wang, C et al. [99] utilized metalens to implement a holographic near-eye 3D display system. Fan, Z.-B et al. [100] implemented a complete near-eye display system using a metalens array as shown in Figure 8b. Due to the large number of metalenses used, NIL was employed to significantly reduce both cost and production time. They proposed an EIA (Elemental Image Array) real-time rendering method combined with a voxel-pixel mapping lookup table, which only requires lookup operations during rendering, achieving a rendering frame rate of 67 FPS while reducing the hardware cost required for rendering. By integrating the metalens with EIA rendering, they constructed an AR system with an FOV of 2.29°, DOF ranging from 100 to 375 mm, and an angular resolution of 14.4 PPD (pixels per degree). These examples demonstrate that metalenses hold substantial potential for diverse applications.

## 5. Fabrication Methods

As previously mentioned, the geometric parameters of meta-atoms are typically on the same order of magnitude as the operating wavelength. Therefore, fabricating metalenses composed of thousands to millions of meta-atoms requires extremely high-precision manufacturing techniques. Currently, metalens fabrication primarily relies on DUV photolithography or electron-beam lithography (EBL). These methods involve altering the chemical properties of a photoresist to define the desired patterns, followed by development, resist removal, thin-film deposition, and etching processes to realize the metalens structures.

These techniques achieve nanometer-scale resolution and are widely used in nanodevice fabrication. However, there are several limitations when applying these methods to metalens production. DUV photolithography is a mask-based technique; thus, fabricating different types of metalenses requires producing corresponding high-precision masks, which increases manufacturing cost and process complexity. On the other hand, EBL is a maskless lithography method, in which a focused electron beam directly exposes the photoresist, locally changing its chemical properties. After development, the desired nanostructures are formed on the photoresist.

Although the electron beam’s extremely short wavelength offers very high theoretical resolution, practical challenges remain. For densely packed patterns and high exposure doses, proximity effects can degrade resolution and pattern fidelity, reducing the accuracy and precision of the fabricated nanostructures. Additionally, EBL operates via serial point-by-point scanning with limited scan areas, resulting in long processing times and making it unsuitable for large-area metalens fabrication. Furthermore, the high cost of equipment and processing poses significant barriers to the commercial scalability of metalenses, indirectly limiting their broader research applications. Therefore, further research into scalable and cost-effective fabrication methods is necessary to advance the widespread adoption and commercialization of metalenses.

Currently, researchers have employed novel micro-nanofabrication techniques such as NIL to fabricate metalenses. NIL can be mainly categorized into thermal nanoimprint lithography (T-NIL) and ultraviolet nanoimprint lithography (UV-NIL), both requiring the fabrication of nanostructured templates through a series of processes including photolithography.

During the pattern transfer process, T-NIL involves spin-coating a thermoplastic patternable material onto the substrate surface. The material is then heated to its glass transition temperature, after which the mold is pressed vertically into the softened patternable material. The mold is held under controlled temperature and pressure for a certain duration to transfer the pattern into the material, followed by cooling and demolding to complete the pattern transfer. Due to the relatively long heating and cooling times, T-NIL suffers from extended overall fabrication cycles.

For large-scale metalens fabrication demanding higher efficiency and precision, UV-NIL has been increasingly adopted because of its advantages such as rapid curing at room temperature. In the process of UV-NIL, a UV-sensitive patternable material is spin-coated on the substrate surface, and then pressed with relatively low pressure to fill the nanostructures in the mold. Subsequent UV exposure for a designated time cures the patternable material, thus completing the pattern transfer. Since UV light must penetrate through the mold to cure the material underneath, the mold requires high ultraviolet transmittance.

Nanoimprint lithography can replace the photolithography step in conventional fabrication processes. Although photolithography and other processes are still necessary to fabricate reusable nanostructured templates, these templates significantly reduce the unit cost of producing individual metalenses in mass production. Fan, Z.-B et al. [100] demonstrated the fabrication of large-area metalens arrays using nanoimprint lithography. Dirdal, C et al. [101] employed UV-NIL combined with deep silicon etching to fabricate near-infrared metalenses composed of meta-atoms with a height of 1200 nm, confirming the optical properties of the metalens through focusing experiments. Since template removal after polymer curing inevitably introduces stress-induced deformation and potential pattern damage, Choi, H et al. [19] proposed the use of water-soluble PVA molds to reduce pattern deformation risks during demolding. By mixing TiO_2_ and resin solvents, they prepared a TiO_2_-containing photosensitive nanocomposite resin (PER), and ultimately fabricated metalenses composed of meta-atoms with an aspect ratio up to 6:1 using UV-NIL and related processes. Although the PVA molds reduce deformation risk during demolding, this advantage is achieved at the expense of mold reusability. Kim, J et al. [102] successfully fabricated reusable high-precision replica molds using hard polydimethylsiloxane (h-PDMS) and other materials, achieving high-fidelity and low-cost replication of 12-inch master molds. Combining UV-NIL with TiO_2_ atomic layer deposition (ALD), they produced resin metalenses with high refractive index and centimeter-scale apertures, demonstrating a phase conversion efficiency as high as 89.6% in the visible spectrum and showcasing their practical potential in near-eye display systems. These cases demonstrate that low-cost and large-scale fabrication of metalenses is achievable.

## 6. Discussion

Starting from the generalized Snell’s law, this article introduces the fundamental principles of metalenses, followed by a discussion of various strategies for achieving achromatic performance based on these principles. A series of representative examples demonstrates the significant progress made in chromatic aberration correction using metalenses. However, challenges remain in aspects such as aperture size, NA and efficiency, which are inherently tied to the underlying mechanisms of achromatic design. As mentioned in the literature, researchers have attempted to overcome these limitations through approaches such as designing multilayer metalenses and using deep learning to train achromatic models. Nevertheless, these methods bring new challenges, including increased fabrication complexity and the need for large training datasets.

In addition, metalenses still face other limitations, such as high production costs and long fabrication cycles. Despite these challenges, the ultra-thin form factor and powerful light manipulation capabilities of metalenses remain unmatched by conventional lenses. Hybrid systems that combine the advantages of traditional refractive lenses and metalenses hold promise for further breakthroughs. Traditional optical systems often require multiple lens elements to correct various aberrations. Thanks to the high design freedom and wavefront engineering capabilities of metalens, it is feasible to tailor them to compensate for the aberrations introduced by refractive lenses, thereby reducing the number of optical elements in the system.

Furthermore, with the advancement of computational optics and the rapid increase in computing power, the training time required for deep learning-based achromatic models is expected to decrease significantly. The continued development of nanoimprint lithography also offers promising routes for large-scale fabrication of metalenses. As discussed earlier, multilayer metalenses can offer larger phase dispersion compensation, opening up new possibilities for broadband achromatic metalens designs. Advances in fabrication technologies are expected to further support the practical realization of such multilayer structures.

As introduced in the principles section, metasurfaces based on geometric phase are inherently polarization-sensitive, limiting their use to polarized incident light and causing partial energy loss. However, this polarization dependence provides additional degrees of freedom for optical design. For instance, in holographic metasurfaces [103], geometric phase engineering enables the generation of distinct holographic patterns under different polarization states. Moreover, leveraging this polarization sensitivity, Feng, X et al. [104] have designed arrays of metalenses with identical focal lengths but selective responses to different polarization states, achieving a broadband polarization and wavefront detection system. Geometric phase can also be used to design metasurface devices with complex functionalities, such as those used in optical communication and optical differentiators [105]. Du, X et al. [106] employed geometric phase methods to design a metalens that generates both zero-order and higher-order Bessel beams under different polarization inputs, enabling achromatic imaging with an extended DOF. This design exceeded the size limitations of conventional broadband achromatic metalenses. By switching the polarization state and applying an intensity subtraction process, high-quality full-color imaging across the visible spectrum can be achieved. These examples demonstrate that geometric phase engineering offers a promising pathway for designing advanced and multifunctional optical systems. Additionally, the performance of metalenses varies significantly depending on the constituent materials, highlighting the importance of not only theoretical and design innovations but also materials optimization. In summary, with the continued development of adjacent fields, metalenses are expected to overcome existing theoretical barriers and achieve significant advancements.

## Figures and Tables

**Table 1:** Summary of Metalenses.

Material	Wavelength	NA	Fabrication	Focusing Efficiency	Ref.
Si	1000 nm–1700 nm	0.15	Not mentioned	64%	[1]
Si	3500 nm–4750 nm	0.45	EBL	45% (4250 nm)	[40]
TiO_2_	490 nm–550 nm	0.20	Electron beam deposition.	Not mentioned	[9]
GaN	400 nm–660 nm	0.15	Mask transfer and etching process	40%	[41]
SiN	400 nm–700 nm	0.27	EBL	50%	[42]
